# HbA1c reconsidered: glycated haemoglobin in the era of dynamic erythrocyte glucose metabolism

**DOI:** 10.1007/s00592-026-02800-7

**Published:** 2026-09-19

**Authors:** Ming-Yu Hsieh

**Affiliations:** 1 Division of Pediatric Surgery, Department of Surgery, Chung Shan Medical University Hospital, Taichung, Taiwan; 2 Center for Evidence-Based Medicine, Chung Shan Medical University Hospital, Taichung, Taiwan; 3 School of Medicine, Chung Shan Medical University, Taichung, Taiwan

**Keywords:** HbA1c, Erythrocyte glucose transport, GLUT1, Diabetes monitoring, Personalized medicine, Hypoxia

## Abstract

Glycated haemoglobin (HbA1c) has been the standard biomarker of long-term glycaemic control for four decades. It rests on a tacit assumption: that non-enzymatic haemoglobin glycation is governed chiefly by mean plasma glucose, with erythrocyte intracellular glucose in passive equilibrium with plasma. This requires erythrocyte glucose uptake—and thus glucose transporter 1 (GLUT1) expression—to be effectively constant. The 2026 demonstration that chronic hypoxia upregulates erythrocyte GLUT1 approximately twofold and per-cell glucose uptake approximately threefold, together with evidence that the band 3 N-terminus acts as a bidirectional metabolic switch responsive to haemoglobin oxygenation, challenges this assumption. We propose that HbA1c is better understood as integrating erythrocyte intracellular glucose exposure, a quantity predicted to diverge from plasma glucose when the erythrocyte oxygen environment or red cell turnover is altered. We review reported discordance in high-altitude populations, obstructive sleep apnoea, cyanotic congenital heart disease, critical illness and chronic kidney disease, and propose testable predictions and a research roadmap. The framework is intended to complement, not replace, established explanations such as altered red cell survival, erythropoiesis, iron status and analytical interference. No human study has yet measured erythrocyte GLUT1 expression, intracellular glucose exposure and HbA1c discordance concurrently; the framework is therefore presented as a hypothesis for prospective testing rather than as a basis for changing clinical practice.

## Introduction: an underexamined assumption underlying HbA1c

Glycated haemoglobin (HbA1c) is among the most widely used biomarkers in modern medicine. First validated for diabetes monitoring in the late 1970s [1] and codified as a diagnostic criterion by the American Diabetes Association in 2010 [2], HbA1c is now measured several hundred million times annually worldwide. Treatment intensification, drug approvals, cardiovascular outcome trials, public-health diabetes surveillance, and individual prognosis all rest on the interpretation of this single number.

The molecular basis is straightforward: glucose binds non-enzymatically to the N-terminal valine of the β-chain of haemoglobin A0, forming a Schiff base that undergoes Amadori rearrangement to a stable ketoamine [3]. The reaction is irreversible during the erythrocyte’s lifespan. The amount of glycated haemoglobin accumulated by an individual red cell is therefore the time-integral of its intracellular glucose exposure over its circulating life.

Translating this molecular picture into a clinically usable biomarker requires three simplifying assumptions:

- Erythrocyte intracellular glucose concentration approximates plasma glucose concentration.
- Erythrocyte lifespan is reasonably uniform (~ 120 days).
- Glycation rate constants are similar across individuals.

Decades of clinical experience have shown that all three assumptions can fail. Inter-individual variation in HbA1c at any given mean blood glucose is well documented [4]; the same true mean glucose may yield HbA1c values differing by more than 1% point (approximately 11 mmol/mol), corresponding to estimated mean glucose differences exceeding 60 mg/dL [5]. Mechanistic models incorporating subject-specific red cell age and glycation kinetics have improved the situation modestly [5, 6], but unexplained variance remains substantial.

This perspective argues that a fourth, hitherto-unrecognised source of variability has now become visible: erythrocyte glucose uptake itself is not constant. The 2026 demonstration by Martí-Mateos and colleagues [7] that chronic hypoxia upregulates erythrocyte GLUT1 protein abundance approximately twofold and per-cell glucose uptake approximately threefold, together with the broader recognition that band 3 acts as a bidirectional regulator of glycolytic flux [8, 9], invites re-examination of a fundamental assumption underlying HbA1c. If GLUT1-mediated transport is a regulated variable rather than a fixed constant, then the quantity that HbA1c integrates may differ from what has conventionally been assumed.

## The dynamic erythrocyte: a 2026 view

### Erythrocyte GLUT1 as a regulated variable

The Martí-Mateos et al. study used murine chronic hypoxia (8% O₂ × 4 weeks) combined with positron emission tomography, isotopic glucose flux analysis, phlebotomy, and red cell transfusion experiments to identify red cells as a primary glucose sink [7]. Key quantitative findings:

- Approximately 70% of the hypoxia-induced increase in systemic glucose uptake could not be accounted for by visceral organ uptake.
- Newly synthesised red cells produced under hypoxic erythropoiesis showed approximately 2-fold higher GLUT1 protein per cell.
- Per-cell glucose uptake rate increased approximately 3-fold and persisted ex vivo under normoxic conditions—a stable cellular phenotype, not an acute signalling response.
- Manipulating red cell number through phlebotomy or transfusion directly altered plasma glucose, *indicating that red cells are both necessary and sufficient for the glycaemic effect in this model*.

In parallel, Issaian et al. characterised the band 3 N-terminus as a bidirectional metabolic switch [8]. Under deoxygenation, deoxyhaemoglobin competitively displaces glycolytic enzymes (GAPDH, PFK, aldolase) from band 3, releasing them into the cytosol and accelerating glycolysis with downstream 2,3-DPG accumulation. Under oxygenation or oxidant stress, glycolytic enzymes themselves bind band 3, partially inhibiting glycolysis and redirecting flux through the pentose phosphate pathway to generate NADPH for antioxidant defence. Storage biology experiments by Reisz et al. confirmed this switching behaviour experimentally: hyperoxic storage produced higher methaemoglobin accumulation and elevated PPP flux throughout the storage period [9].

### Single-cell heterogeneity

Recent single-cell imaging using the fluorescent glucose analogue 2-NBDG has revealed substantial heterogeneity in erythrocyte glucose uptake both within and between individuals [10]. The authors of that work explicitly note that this transporter-level variation will affect HbA1c formation, and propose that more personalised diagnostic strategies may be warranted. Their work was published independently of the chronic hypoxia framework, but the two observations converge on the same conclusion: erythrocyte glucose handling is not a fixed background variable.

## Reinterpreting HbA1c

**Fig. 1:**
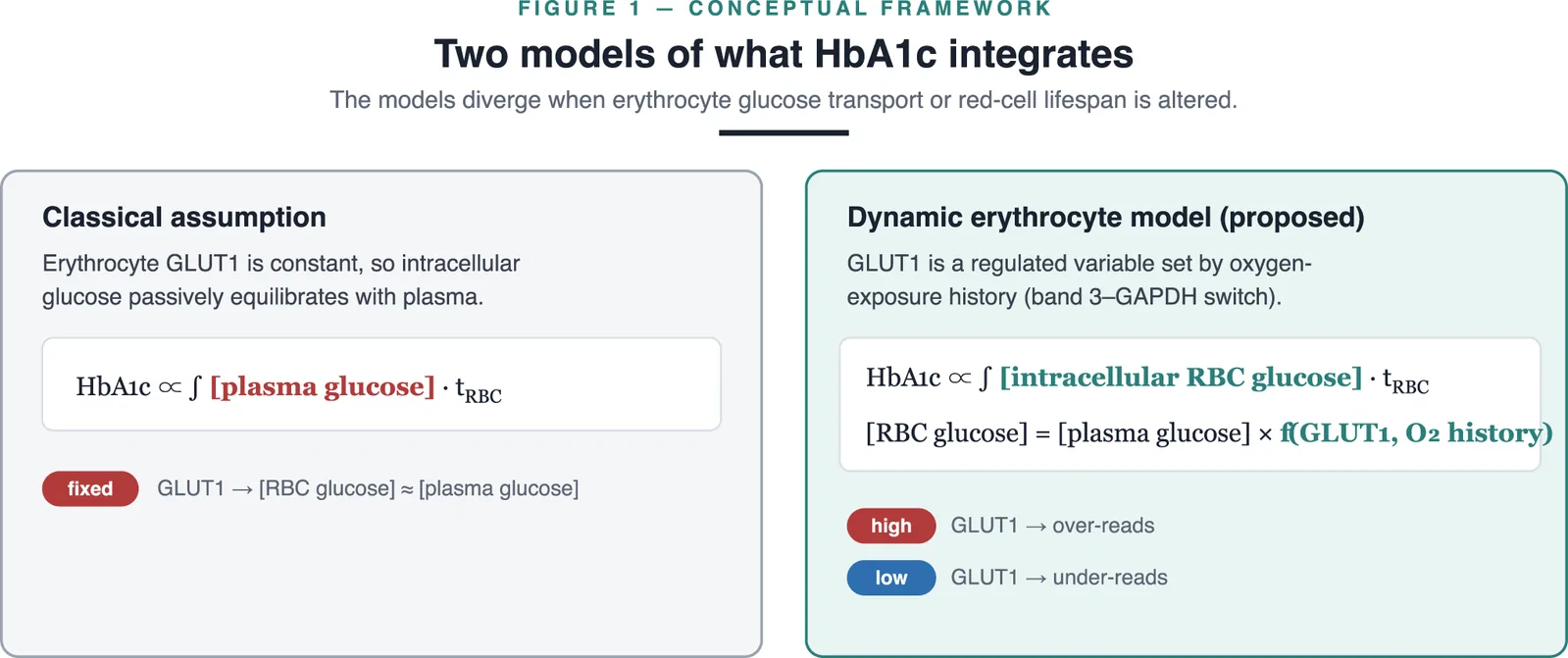
Two models of the quantity integrated by HbA1c. Under the classical assumption (left), erythrocyte GLUT1 is constant and intracellular glucose equilibrates passively with plasma, so HbA1c approximates the time-integral of plasma glucose scaled by red-cell lifespan. In the dynamic model proposed here (right), GLUT1 is a regulated variable set by oxygen-exposure history, so the integrated quantity is intracellular rather than plasma glucose; higher GLUT1 would cause HbA1c to over-read and lower GLUT1 to under-read relative to plasma glucose. The two models coincide when erythrocyte glucose transport and red-cell lifespan are stable.

Combining these observations leads to a refined conceptualisation of HbA1c. We propose the following:

We propose that HbA1c is better understood as integrating intracellular erythrocyte glucose exposure — scaled by erythrocyte lifespan and glycation kinetics — rather than the time-integral of plasma glucose alone. Under conditions of stable erythrocyte glucose transport, these two quantities track closely. Under conditions of altered erythrocyte oxygen environment or accelerated red cell turnover, they are predicted to diverge in directionally predictable ways (Fig. 1).

We distinguish three levels of evidence in what follows: observations that are well established (the dependence of HbA1c on erythrocyte lifespan and turnover); mechanistic inferences drawn from experimental models (the regulation of erythrocyte GLUT1 by oxygen exposure); and testable hypotheses that follow from combining the two (the resulting effect on HbA1c interpretation in specific human populations). The last of these has not yet been demonstrated directly in humans. This proposition is additional to, and does not displace, the established determinants of HbA1c discordance discussed in “Established determinants of HbA1c discordance”.

Formally, the steady-state HbA1c concentration follows:

$$HbA1c \propto k\_glycation \times [glucose]\_intracellular\:\times\:\tau\_RBC$$

Where k_glycation is the second-order rate constant for haemoglobin glycation, [glucose]_intracellular is the time-averaged intracellular glucose concentration, and τ_RBC is the mean red cell lifespan. The traditional simplification asserts that [glucose]_intracellular ≈ [glucose]_extracellular, which holds when GLUT1-mediated transport is rapid relative to glycation and when GLUT1 expression is uniform. In the dynamic erythrocyte framework, this approximation fails:

$$\begin{aligned} &[glucose]\_intracellular = f(GLUT1\_expression,\\ & \quad glycolytic\_flux, band\mathrm{3-}GAPDH\: binding\: state)\end{aligned}$$

The first variable (GLUT1 expression) governs influx; the second (glycolytic flux) governs efflux through metabolism; the third (band 3 state) modulates both. Each of these varies with the erythrocyte’s oxygen exposure history.

## Established determinants of HbA1c discordance

Discordance between HbA1c and directly measured glycaemia is not a new observation, and a substantial literature already accounts for much of it. Any additional mechanism must therefore be positioned explicitly against these established explanations.

 Erythrocyte lifespan and turnover. The most extensively documented determinant is variation in red cell survival. Using biotin labelling, Cohen et al. found mean red cell age to range from 39 to 56 days in people with diabetes and from 38 to 60 days in non-diabetic controls — variation sufficient to produce clinically important differences in HbA1c at the same mean glucose [4]. Mechanistic modelling by Malka et al. subsequently indicated that between-patient differences in derived mean red cell age could account for the glucose-independent variation in HbA1c, and that personalising estimates on this basis reduced errors by more than half [5]. Models incorporating red cell lifespan have since been proposed for routine interpretation [6].

Erythropoiesis and erythropoiesis-stimulating agents. The rate of red cell production is a further independent determinant. In 102 haemodialysis patients, Rasche et al. found ESA dose to be inversely correlated with HbA1c, an increase of 10,000 IU per week corresponding to an estimated fall of approximately 0.6% points; the rise in reticulocyte count was likewise inversely correlated with the change in HbA1c [11]. Notably, the same ESA exposure had no comparable effect on fructosamine, indicating that the effect operates through the erythrocyte compartment rather than through glycaemia itself. In dialysis populations, therefore, a substantial part of the HbA1c underestimate is attributable to shortened red cell survival together with ESA-driven erythropoiesis, and glycated albumin has been proposed as an alternative on that basis [12].

 Iron status, transfusion, plasma volume and analytical interference. Iron deficiency and iron repletion, transfusion of donor erythrocytes with a different age distribution, shifts in plasma volume, haemoglobin variants, and assay-specific analytical interference are all recognised causes of misleading HbA1c results and have been reviewed in detail elsewhere [13].

Relation to the present framework. These mechanisms share a common feature: they act principally on the number, age distribution and survival of circulating erythrocytes — that is, on τ_RBC and on the population of cells available for glycation. The framework proposed here concerns a different term of the same relationship: the intracellular glucose concentration to which those cells are exposed. The two are therefore complementary rather than competing, and we do not propose that dynamic erythrocyte glucose transport replaces established explanations. In the clinical populations considered in the following section, established mechanisms plausibly account for a substantial — and possibly the major — share of the observed discordance. The contribution of regulated erythrocyte glucose uptake is at present an additional candidate term whose magnitude in humans has not been quantified.

## Empirical evidence: HbA1c discordance in specific populations

Several patient populations have long shown HbA1c values that diverge from expected glycaemic patterns. We argue that the dynamic erythrocyte framework provides a unified explanation (Fig. 1). Table 1 summarises these populations together with the established mechanisms already recognised in each.

**Table 1:** Populations in which HbA1c has been reported to diverge from other measures of glycaemia, with established and proposed contributing mechanisms

Population	Established mechanisms	Proposed additional contribution	Reported direction of HbA1c
High-altitude residents	Polycythaemia and haemodilution; altered red-cell survival	Altered GLUT1-mediated glucose uptake	Discordant with FPG/GA
Obstructive sleep apnoea	Intermittent hypoxia and insulin resistance; mixed red-cell cohort	Partial hypoxic reprogramming of glucose uptake	Elevated relative to CGM-derived glucose
Cyanotic congenital heart disease	Chronic hypoxia; HbF; erythrocytosis	Developmental change in erythrocyte glucose handling	Relatively low
Critical illness	Hyperoxia/hypoxia swings; transfusion; accelerated turnover	Suppression of the erythrocyte glucose sink	Bidirectional
Chronic kidney disease	Shortened red-cell lifespan; ESA exposure; iron status	S1P-driven metabolic reprogramming	Underestimates glycaemia

### High-altitude populations: a natural experiment

Multiple studies in Tibetan and Andean highlanders have documented HbA1c values that do not track expected relationships with plasma glucose [14–16]. The most directly relevant peer-reviewed evidence is a retrospective matched comparison of people with type 2 diabetes in Lhasa (3650 m) and at sea level in Beijing [17]. Matched for age, sex and fasting serum glucose, the high-altitude group had substantially higher HbA1c (11.9 ± 2.7% [107 mmol/mol] versus 10.4 ± 1.9% [90 mmol/mol]; *P* = 0.001) despite lower postprandial glucose (12.9 ± 3.9 versus 15.0 ± 3.9 mmol/l; *P* = 0.003). HbA1c correlated positively with red cell count (*r* = 0.297; *P* = 0.001), haemoglobin and haematocrit, and in stepwise regression red cell count remained independently associated with HbA1c after adjustment for postprandial glucose, BMI and sex. Notably, those authors argued that this association is unlikely to be explained by altered red cell survival, citing evidence that erythrocyte lifespan is normal in high-altitude polycythaemia.

 A larger population-based study of 1,401 Tibetan adults, using the oral glucose tolerance test as the reference standard, similarly found that red cell count—but not haemoglobin—was independently associated with HbA1c after adjustment for age and fasting or 2-hour glucose (β = 0.140; *P* < 0.001), and that the optimal HbA1c cut-off for detecting diabetes differed in high-altitude polycythaemia [18].

Both studies indicate that, in high-altitude populations, HbA1c departs from concurrently measured glucose in association with erythrocyte indices—particularly red cell count—rather than with glycaemia alone. Neither, however, identifies which erythrocyte-level mechanism is responsible, and haemodilution, altered red cell survival and altered glucose handling remain competing explanations.

A Tibetan population study has likewise demonstrated that the optimal HbA1c cutoff for detecting abnormal glucose metabolism differs between high- and low-altitude residents [19], and the authors recommend population-specific thresholds. The framework advanced here offers a possible mechanistic explanation that requires validation.

### Obstructive sleep apnoea: a complex case

HbA1c is positively associated with OSA severity in a dose-response manner [20, 21]. A 2139-patient study showed odds ratios for HbA1c > 6.0% (42 mmol/mol) rising from 1.0 (AHI < 5) to 2.96 (AHI ≥ 50) after adjustment for traditional confounders [20]. The conventional interpretation attributes this to intermittent hypoxia-driven insulin resistance.

The dynamic erythrocyte framework adds nuance. OSA partially activates erythrocyte hypoxic reprogramming—not enough to fully replicate the chronic high-altitude phenotype (only ~ 2% of OSA patients develop overt polycythaemia [22]), but enough to alter erythrocyte glucose handling. Partial GLUT1 upregulation may increase intracellular glucose exposure relative to extracellular, contributing to HbA1c elevation independent of true plasma hyperglycaemia. The proportion attributable to insulin resistance versus erythrocyte-level glycation enhancement has not been disentangled.

This has potential clinical implications. The American Diabetes Association threshold of HbA1c ≥ 6.5% (48 mmol/mol) for diabetes diagnosis may overestimate diabetes prevalence in OSA populations if a substantial fraction of HbA1c elevation reflects erythrocyte-level rather than systemic glucose dysregulation. CGM-based assessment, which bypasses erythrocyte processing entirely, would help quantify the discrepancy. This remains a hypothesis requiring direct comparison against CGM- or OGTT-based diagnosis rather than a demonstrated phenomenon.

### Cyanotic congenital heart disease: a developmental layer

Cyanotic CHD children represent perhaps the clearest natural model of chronic erythrocyte hypoxic adaptation. Versmold et al. demonstrated decades ago that 2,3-DPG and P50 rise significantly in CCHD patients after the foetal-to-adult haemoglobin switch (> 3 months age) [23], and Gidding and Stockman characterised the high-haematocrit/low-EPO phenotype [24]. These observations describe an erythrocyte population with substantially altered metabolic state.

If the framework is correct, CCHD children should exhibit HbA1c values that progressively decouple from true plasma glucose as the haemoglobin switch completes. Hypoglycaemia during fasting (originally reported by Haymond et al. in 1979 [25]) and concurrently relatively low HbA1c could hypothetically share an erythrocyte-mediated contribution: erythrocyte-level glucose consumption exceeding plasma supply. We are not aware of any study that has directly measured HbA1c discordance with mean glucose in CCHD children stratified by HbA0/HbF ratio—a gap that remains to be addressed.

### Critical illness: a bidirectional perturbation

Critical illness adds multiple perturbing factors: sustained hyperoxia (which we have proposed elsewhere may suppress the glucose sink; manuscript in preparation), neocytolysis (accelerating red cell turnover [26]), and frequent transfusion (introducing donor erythrocytes with different histories). HbA1c interpretation in critically ill or recently-discharged-from-ICU patients is widely recognised as unreliable. The framework here provides a mechanistic basis: the ICU environment alters every term in the HbA1c equation simultaneously, in directions that are not easily predicted without measurement of *erythrocyte characteristics themselves*.

### Chronic kidney disease: a documented underestimate

CKD patients on dialysis exhibit HbA1c values that systematically underestimate glycaemia, and glycated albumin has been proposed as the preferred alternative [12]. The conventional explanation invokes shortened red cell lifespan from uraemia and EPO deficiency. The dynamic framework adds that the angiotensin-SphK1-S1P axis—described in CKD by Xie et al. [27]—directly reprograms erythrocyte glucose metabolism toward glycolysis, paradoxically lowering steady-state intracellular glucose available for glycation despite preserved plasma levels. This is a population in which traditional and dynamic explanations converge to predict the same direction of HbA1c bias, both contributing.

## Testable predictions

The framework generates several falsifiable predictions amenable to clinical investigation:

1. In any population with altered chronic oxygen environment (high altitude, OSA, CCHD, COPD, ICU survivor), the discrepancy between HbA1c-estimated and CGM-measured average glucose will correlate with erythrocyte GLUT1 expression as measured by flow cytometry.
2. Glycated albumin will track CGM-measured glucose more faithfully than HbA1c in patients with high-altitude residence, severe OSA, polycythaemic CHD, and CKD.
3. Therapeutic interventions that alter erythrocyte oxygen environment—initiation of CPAP in OSA, surgical correction of CCHD, descent from altitude, transition from ICU to ward—would be expected to produce changes in HbA1c that lag behind changes in true glucose by approximately one red cell turnover (~ 3–4 months), as the existing red cell cohort is progressively replaced.
4. A composite erythrocyte metabolic biomarker—we propose the Red Cell Hypoxic Metabolic Index (RHMI), incorporating GLUT1 expression, ex vivo glucose uptake, 2,3-DPG, and methaemoglobin, the components of which are specified in a study protocol in preparation—might serve as a personalised correction factor for HbA1c interpretation. Patients with high RHMI (hypoxia-adapted erythrocytes) would be expected to require downward correction of HbA1c-derived estimated average glucose; patients with low RHMI (hyperoxia-suppressed erythrocytes) upward correction.

## Clinical implications

The implications set out below would follow if the framework were substantiated. They are presented as directions for investigation rather than as recommendations for current clinical practice.

### Population-specific HbA1c thresholds

The Tibet Plateau studies have already moved in this direction with empirical recalibration of HbA1c cutoffs [19]. The framework advanced here raises the question of whether such recalibration should be examined more systematically:

- High-altitude residents (> 2500 m for > 3 months): whether the diagnostic threshold requires recalibration warrants prospective evaluation.
- Severe OSA (AHI > 30) prior to CPAP: the adequacy of HbA1c alone for diagnosis should be assessed against CGM-derived measures.
- Unrepaired CCHD: the informativeness of HbA1c for glycaemic status requires direct assessment before alternatives are adopted.
- Post-ICU survivors during first 3–4 months: the extent to which HbA1c reflects pre-ICU glycaemia requires quantification.

### Choice of monitoring biomarker

In specific populations, glycated albumin or 1,5-anhydroglucitol may better reflect glycaemic state. CGM, which directly measures interstitial glucose, bypasses erythrocyte processing entirely and provides a valuable reference for longitudinal glucose exposure, although it has its own measurement and sampling limitations. Future monitoring frameworks may need to consider erythrocyte phenotype as one determinant of biomarker choice.

### Diabetes complications: a re-examination

HbA1c predicts microvascular complications because diabetic retinopathy, nephropathy, and neuropathy occur in cells (retinal pericytes, renal mesangial cells, peripheral neurons) that cannot adjust GLUT transporter expression in response to hyperglycaemia [28]. Traditionally, erythrocytes were considered to fall in the same category—hence HbA1c’s predictive value. In the dynamic framework, this equivalence breaks down: erythrocytes can adjust GLUT1, but the affected complication-prone cells cannot. The prediction is that in populations where the erythrocyte-vulnerable-cell coupling is disrupted (chronic hypoxia, ICU), HbA1c may lose some of its predictive power for microvascular complications. Discordances of this kind have been reported [29]; the framework offers one possible mechanistic contribution, among others, to their explanation.

### Therapeutic erythrocyte modulation

The NIH-sponsored trial NCT04137692 testing red blood cell exchange transfusion for GLUT1 deficiency syndrome explicitly relies on the concept that erythrocytes are a manipulable glucose handling compartment. This programme establishes a related biological principle—that erythrocytes are a manipulable glucose-handling compartment—rather than validating the proposed effect on HbA1c. If therapeutic erythrocyte modulation proves effective in any disease context, HbA1c would be expected to become less reliable as a monitoring tool for treatment response—because the intervention by design changes the relationship between plasma and intracellular erythrocyte glucose.

## Limitations and caveats

Several constraints qualify the framework.

First, the most direct evidence for dynamically regulated GLUT1 expression remains a murine chronic hypoxia model. Whilst single-cell human erythrocyte studies [10] and high-altitude human cohort data [17, 18] are consistent with the framework, no clinical study has to date simultaneously measured erythrocyte GLUT1 expression, intracellular glucose exposure and HbA1c–glucose discordance within the same human cohort, and direct measurement of GLUT1 across the populations discussed here has not been undertaken at scale. The framework therefore remains a biologically plausible hypothesis awaiting prospective validation. An ICU observational study is currently in preparation.

Second, the framework does not eliminate the value of HbA1c. In healthy adults with stable oxygen environment, the constancy assumption holds approximately, and HbA1c remains an excellent biomarker. The argument is for refinement, not replacement.

Third, the quantitative magnitude of GLUT1-driven HbA1c distortion in human populations has not been precisely measured. Whilst the available high-altitude data suggest effects of potential clinical relevance [17, 18], these remain observational and cannot yet establish a transport-level mechanism, and we cannot yet assign a numerical correction factor with confidence.

Fourth, the foetal-to-adult haemoglobin switch introduces additional complexity in paediatric populations. HbF does not bind 2,3-DPG to the same extent as HbA [23], and the glycation rate of HbF1c versus HbA1c may differ. The framework’s predictions for paediatric CCHD therefore require age-specific calibration.

## A research roadmap

Moving from a conceptual model to clinical application requires evidence that does not presently exist. We outline four sequential steps.

Step 1 Concurrent measurement in humans. The most informative single study would measure, within the same individuals, erythrocyte GLUT1 expression by flow cytometry, mean glucose by continuous glucose monitoring, HbA1c and glycated albumin, in cohorts selected for contrasting chronic oxygen exposure — long-term high-altitude residents, patients with severe obstructive sleep apnoea, and patients with chronic kidney disease — each with matched controls. Critically, the established determinants set out in Sect.  4 (red cell survival, reticulocyte indices, iron status and erythropoiesis-stimulating agent exposure) should be measured concurrently, so that any residual association attributable to glucose transport can be distinguished from them.
Step 2 Mechanistic confirmation ex vivo. In the same participants, ex vivo glucose uptake, intracellular glucose exposure, band 3–glycolytic enzyme association and 2,3-DPG would test whether transport-level regulation, rather than altered cell survival alone, accounts for any discordance identified in Step 1.
Step 3 Longitudinal and interventional testing. Prediction 3 (Sect.  6) is directly testable. Following initiation of CPAP in obstructive sleep apnoea, surgical correction of cyanotic congenital heart disease, or descent from altitude, serial measurement of HbA1c against continuous glucose monitoring over approximately one red cell turnover (three to four months) would determine whether the predicted lag is observed.
Step 4 Index construction and validation. Only if Steps 1 to 3 prove supportive would construction of a composite index such as the RHMI be warranted, followed by prospective testing of whether it improves the accuracy of HbA1c-derived estimates of mean glucose. Paediatric populations would require separate calibration stratified by HbF/HbA0 ratio.

Until at least Steps 1 and 2 have been completed, the framework should be regarded as a hypothesis for testing rather than as a basis for modifying clinical practice.

## Conclusion

HbA1c may usefully be conceptualised as reflecting erythrocyte intracellular glucose exposure rather than plasma glucose alone, with the cellular biology that this implies. For four decades this distinction had limited clinical consequence because the erythrocyte was assumed—reasonably, given the tools available—to be in passive equilibrium with plasma. The 2026 demonstration that erythrocytes are active, regulated glucose sinks calls that assumption into question.

The implications are not narrowly methodological. HbA1c shapes treatment decisions for hundreds of millions of patients with diabetes, prediabetes, and metabolic risk. Reinterpreting it through the dynamic erythrocyte lens points toward:

- Population-specific diagnostic thresholds.
- Biomarker selection guided by erythrocyte phenotype.
- Reconciliation of long-standing discordances between HbA1c and clinical outcomes.
- Anticipation of new monitoring challenges as erythrocyte-targeting therapies emerge.

The traditional reading of HbA1c served medicine well in an era when the erythrocyte was understood as a passive vessel. As that understanding gives way to one of dynamic metabolic activity, the biomarker that defined modern diabetology requires a parallel update in interpretation. HbA1c remains an essential tool; its interpretation may benefit from explicit consideration of erythrocyte physiology. The framework is offered as a complement to established explanations rather than a replacement for them.

## Data Availability

Not applicable; no original datasets were generated or analysed in this Perspective.

## Declarations

### Use of AI

Large language model tools (Claude, Anthropic) were used to assist with literature synthesis, drafting, and reference verification. All references were checked against primary sources (PubMed; and, for non-indexed preprints, the preprint server—such references are labelled as non-peer-reviewed). The author reviewed and verified all content and citations, and takes full responsibility for the scientific interpretation, the balance of the discussion and the strength of the conclusions presented.

### Preprint

An earlier version of this article has been posted as a preprint on Preprints.org (https://doi.org/10.20944/preprints202607.0826).
